# Extracellular vesicles have variable dose‐dependent effects on cultured draining cells in the eye

**DOI:** 10.1111/jcmm.13505

**Published:** 2018-02-07

**Authors:** Saray Tabak, Sofia Schreiber‐Avissar, Elie Beit‐Yannai

**Affiliations:** ^1^ Clinical Biochemistry and Pharmacology Department The Faculty of Health Sciences Ben‐Gurion University of the Negev Beer‐Sheva Israel

**Keywords:** Extracellular vesicles, Wnt, Signaling, Trabecular meshwork, Exosomes, Non pigmented ciliary epithelium

## Abstract

The role of extracellular vesicles (EVs) as signal mediators has been described in many biological fields. How many EVs are needed to deliver the desired physiological signal is yet unclear. Using a normal trabecular meshwork (NTM) cell culture exposed to non‐pigmented ciliary epithelium (NPCE)–derived EVs, a relevant model for studying the human ocular drainage system, we addressed the EVs dose–response effects on the Wnt signaling. The objective of the study was to investigate the dosing effects of NPCE‐derived EVs on TM Wnt signaling. EVs were isolated by PEG 8000 method from NPCE and RPE cells (used as controls) conditioned media. Concentrations were determined by Tunable Resistive Pulse Sensing method. Various exosomes concentration were incubated with TM cells, for the determination of mRNA (β‐Catenin, Axin2 and LEF1) and protein (β‐Catenin, GSK‐3β) expression using real‐time quantitative PCR and Western blot, respectively. Exposure of NTM cells for 8 hrs to low EVs concentrations was associated with a significant decreased expression of β‐Catenin, GSK‐3β, as opposed to exposure to high exosomal concentrations. Pro‐MMP9 and MMP9 activities were significantly enhanced in NTM cells treated with high EV concentrations of (X10) as compared to low EV concentrations of either NPCE‐ or RPE‐derived EVs and to untreated control. Our data support the concept that EVs biological effects are concentration‐dependent at their target site. Specifically in the present study, we described a general dose–response at the gene and MMPs activity and a different dose–response regarding key canonical Wnt proteins expression.

## Introduction

EVs are nanoscale secreted membrane vesicles, derived from the luminal membrane of multivesicular bodies (MVBs). Following the fusion of the MVBs with the cell membrane, these vesicles are released to the extracellular medium 1. EVs carry coding and non‐coding RNA, single strand DNA, lipids, proteins and antigen‐presenting molecules 2. Today, it is well accepted that beyond the classical secretory and exocytic pathways, EVs in general and exosomes in particular play an important role in cell:cell communications 3. EVs function in normal physiology maintaining general homeostasis, and are part of the cell response under disease pathogenesis. Researchers have successfully demonstrated the ability of EVs to manipulate recipient cells in close and distant tissues by the delivery of their biological cargo 4. In addition, EVs are capable to deliver biological messages by releasing active ligands from their surface or adhere and activate the recipient cells receptors 5.

The delivery of biological signals by EVs has been under research in almost all the biological fields. To name a few of them: cancer 6, inflammation 7, stem cells 8, cardiology 9, endocrinology 10, fertility 11, ophthalmology 12 and others. In human aqueous humour, in particular EVs originating from the ocular drainage system and even the retinal pigment epithelium were reported 13, 14. Recently, an increasing number of paper address the signalling delivery by EVs in parasitology 15, yeasts 7 and plant sciences 6. A general approach for EVs‐mediated signal analysis *in vitro* is to expose cell culture to extracted and characterized EVs. A time‐dependent effect of a known amount of EVs is generally described 16. In some cases, various amounts of EVs were tested for their effects on the targeted cells 2, 17, while in other studies, both EVs concentration and exposure time were analysed 1, 2. In many cases, the amount of EVs used is determined according to their protein content as measured by known methods. More precisely, several researches reported the ratio between the EVs amount in milligrams of protein to the targeted cell number. The development of sophisticated methods and dedicated instruments for EVs measurements such as Tunable Resistive Pulse Sensing (TPRS) and Nanoparticle Tracking Analysis (NTA) enabled the studies stating EVs number and sizes. Beyond the methods used to determine the EVs concentrations and sizes, it is yet unclear what should be the ratio EV/target cell to mimic physiological conditions. A range of EVs concentrations from 0.5 μg protein to 50 μg protein per 1 × 10^4^ cells were published 9, 10, 18, 19. Clearly the “real EVs concentrations” vary from one system to the other depending on tissue and cell types, solid tissues *versus* body fluids. Furthermore, alterations in EVs content were reported under stress condition 8, but changes in EVs secretion numbers under malady are still missing. All these factors might contribute to the effects mediated by EVs. Recently, we published a study on EVs role in mediating signals between the aqueous humour‐producing cells, non‐pigmented ciliary epithelium (NPCE), and the aqueous humour draining cells, the trabecular meshwork 12. Wnts are secreted lipid‐modified signalling proteins that influence multiple processes, as well as aqueous humour drainage resistance 20. The canonical Wnt signalling pathway which exists in trabecular meshwork cells 14 is a critical regulator of intraocular pressure and has a role in extracellular matrix (ECM) expression in the trabecular meshwork cells by matrix metalloproteinases (MMPs) activity regulation 12, 13, 17, 21. Among the different MMPs, the gelatinases: MMP2 and MMP9 were reported to play a key role in trabecular meshwork drainage resistance 21, 22.

Canonical Wnt signalling causes β‐catenin accumulation in a complex with the transcription factor TCF/LEF that regulates target gene expression. In the absence of Wnt, β‐Catenin is targeted for ubiquitination and degradation in the proteasome by phosphorylation through the glycogen synthase‐3 β (GSK‐3β) bound to a scaffolding complex containing Axin. Activation of Wnt signalling leads to inhibition of GSK‐3β activity, resulting in accumulation of cytoplasmic β‐catenin, which becomes available to bind the transcription factor TCF/LEF in the nucleus and to induce target gene expression such as AXIN2 and LEF1 These gene are used as markers of the canonical Wnt pathway 17, 20. We used retinal pigment epithelial cells‐derived EVs as control through the study due to their presence in the human aqueous humour 14 and their uptake by normal trabecular meshwork (NTM) cells 12.

In this research, we aimed at examining dose‐dependent effects of EVs on the Wnt signalling mediation, in an attempt to verify how many EVs are needed to address an EVs‐mediated target cells effects using the same cell culture 12.

## Material and methods

### Cell lines

A human NTM cell line was generously donated by Alcon Laboratories, Fort Worth, TX, USA and maintained in Dulbecco's modified Eagle's medium (DMEM) containing 10% fetal bovine serum (FBS), 2 mM L‐glutamine, 100 μg/ml streptomycin and 100 units/ml penicillin (all from Biological Industries, Kibbutz Beit Ha‐Emek, Israel) in a humidified atmosphere of 95% air and 5% CO2 at 37°C. Human non‐pigmented ciliary epithelial (NPCE) cell lines were kindly supplied by Prof. Miguel Coca‐Prados, Yale University. A human retinal pigment epithelium (RPE) cell line was a gift from Dr. Zeev Dvashi, Kaplan Medical Center, Rehovot, Israel, and cultivated under standard conditions of containing 10% FBS and antibiotics. NPCE and RPE cells were cultured in DMEM depleted of FBS‐derived EV by overnight centrifugation at 100,000 *g*. NPCE and RPE cell lines were cultured in DMEM depleted of FBS‐derived EV. The medium was EV depleted by Beckman Coulter ultracentrifugation, for 14 hrs, 4°C and 100,000x *g*. The supernatant was collected and 2 mM L‐glutamine, 0.1 mg/ml streptomycin, and 100 units/ml of penicillin were added.

### Research model

NTM cells were seeded in 6 mm sterile cell culture dishes (2 × 10^6^ cells in 2 ml per dish). 24 hrs later and 1 hr before treatments, growth medium was replaced with fresh EVs‐depleted medium. Different concentrations of NPCE‐ or RPE‐derived EVs were added to NTM cells (100% confluent) to examine concentration depended communication effects. Different concentrations of EVs were tested as follows: 6.8 x 10^9^ EVs/ml (X1); 13.6 x 10^9^ EVs/ml (X2) and 6.8 × 10^10^ EVs/ml (X10). Following 2 or 8 hrs of incubation with the different EVs, Real‐Time PCR analysis or Western blot and Gel Zymography analysis, respectively, were performed. To elucidate EVs interactions with the NTM cells, we used Image Stream analysis. EVs were incubated with NTM cells for 1, 4 and 24 hrs. Then NTM cells were lysed and analysed as described below. The experiments were performed in duplicates and triplicates for 7AAD and EVs uptake, respectively, and repeated independently three times.

### EVs extraction

EVs have been extracted from NPCE and RPE cells using poly‐ethylene‐glycol (PEG) 8000 method 23 with slight modifications. Cell cultures conditioned medium were collected, centrifuged at 1500 *g* for 15 min. at 4°C, to pellet dead cells and cell debris. Precipitation solution was prepared as follows: 50% PEG‐8000, 0.5 M NaCl, mixed with the conditioned medium 1:5 v/v, respectively, filtered through a 0.22 μm PVDF filter and incubated over night at 4°C. The mixtures were centrifuged at 1500 *g* for 30 min. to pellet the EVs. The pellet containing the EVs was resuspended in PBS and pelleted by ultracentrifugation of 100,000 *g* for 70 min. at 4°C. The final EVs pelleted were suspended in 1 ml PBS and were stored at −80°C till use.

### Tunable Resistive Pulse Sensing (TRPS)

EVs concentration was determined by qNano (Izon Science, Christchurch, New Zealand) instrument, using the Tunable Resistive Pulse Sensing (TRPS) principle 24. This principle enables reception of signal while a single particle transfers through NP150, a membrane with pores of 85–300 nm. To eliminate contaminating debris, exosomal samples were passed through 0.22 μm filters. The apparatus was operated at a voltage of 0.48–0.64 V and a pressure equivalent to 8 cm of H_2_O. The membrane was stretched to 45–47 mm. Polystyrene beads at a concentration of 1 x 10^13^ beads/ml (114 nm; Izon Science) were used to calibrate size and concentration, following the manufacturer's instructions. Samples were diluted 1000‐fold with PBS buffer and measured over 10 min.The movement of the particle through the membrane is being identified as change in the ionic stream causing voltage changes. The power of the signal is proportional to the piratical size. According to the amount of particles and their velocity at specific time, the qNano determines the EVs concentration.

### EVs labelling uptake by NTM cells, an image stream study

To assess the kinetics of EVs internalization and to assess the uptake of different EVs concentrations by NTM cells, labelled EV were incubated with NTM cells 25. NTM cells were seeded in 6‐well plates at a density of 0.5 x 10^6^/well in 6 ml medium. EVs‐containing pellets were resuspended in 1 ml of PBS containing 2.5 μl DiD (1,10‐dioctadecyl‐3,3,30,30‐tetramethyl‐indodi‐carbocyanine,4‐chloro‐benzenesulfonate salt Biotium, Hayward, CA, USA). The EVs‐containing pellet was centrifuged for 70 min. at 100,000 *g* and 4°C to remove unincorporated DiD. After cell adhesion, the cells were cultured with DID‐labelled NPCE‐derived EV (6.8 x 10^9^ EVs/ml) for: 1, 4 or 24 hrs. Then, cells were detached by trypsinization, washed twice with PBS by centrifugation of 600 *g* for 5 min. at 25°C, for each wash. NTM cells contains different DID‐labelled EVs concentrations were resuspended in 300 μl of washing buffer (1 mM EDTA, 2% FBS, 0.01 M PBS). Cellular fluorescence was captured and photographed using an ImageStreamX high‐resolution imaging flow cytometer (Amnis, Co., Seattle, WA, USA). Ten thousand cells for each measurement were excited using 642 nm laser. Following the flow cytometry image capture, samples were gated to attain populations of captured single cell images of living cells that were in focus, to quantify the uptake of EV in a defined region.

### NTM cells viability following incubation with EVs, an image stream study

To evaluate the physiological state of NTM cells, in response to diverse EVs concentrations, Image Stream analysis of cell viability was performed using 7‐Amino‐Actinomycin D (7AAD, ABCAM) fluorescence and 488 nm laser. Following R&D systems protocol and system calibrations, our working concentration of 7AAD was determined as 1 mg/ml in PBS). NTM cells incubated for 24 hrs with EVs were harvested up to 1 × 10^6^ cells/300 μl in washing buffer. After cells were detached by 500 μl trypsin, they were transferred to 500 μl growth medium. Cells were washed twice by adding 1 ml PBS, centrifuging at 300× *g* for 5 min., and then decanting the buffer from pelleted cells. 5 μl of 7AAD staining solution to each sample was added, mixed gently and incubated for 30 min. at 4°C in the dark prior to analysis.

### Real‐time PCR analysis

NTM cells were seeded at density of 2 x 10^6^ cells in 6 mm Petri dishes. After 48 hrs, cells were exposed to different NPCE EVs concentrations (X1, X2 or X10 as described above) for 2 hrs. RPE‐derived EVs or DMEM alone were used as controls. Total RNA was isolated using an EZ‐RNA Kit (Biological Industries, Beth Haemek, Israel) according to the manufacturer's instructions. RNA quality and quantity were assessed at 260 nm using a NanoDrop2000 Spectrophotometer (Thermo Scientific, Waltham, MA, USA). Equal amounts (1 μg) of RNA were reverse‐transcribed in triplicates using qScript cDNA Synthesis kit (Applied Biosystems, Foster City, CA, USA). Changes in mRNA levels for Wnt target genes Axin2, LEF1 and β‐Catenin were determined by real‐time PCR with an Applied Biosystems Real‐Time PCR 7500 system (Applied Biosystems) using TaqMan Fast Advanced Master Mix according to the manufacturer's instructions (Applied Biosystems). The incubation and thermal cycling conditions were as follows: hold for 20 sec. at 95°C, denaturation for 3 sec. at 95°C, annealing and extension for 30 sec. at 60°C. The number of cycles was 40. The threshold cycle was measured as the cycle number at which sample fluorescence increases statistically above background and crossing points for each transcript. Human GAPDH gene was used as endogenous control. The mRNA levels were calculated using Expression Suite Software v 1.0.3 (Applied Biosystems).

### Western blot analysis

Following incubation of NTM cells with NPCE‐ or RPE‐derived EVs in different concentrations, and NTM with no treatment as control, the NTM cells were lysed with buffer containing 20 mM HEPES (pH 7.4), 150 mM NaCl, 1 mM EGTA, 1 mM EDTA, 10% glycerol, 1 mM MgCl_2_, 1% Triton x‐100 and 10 μl of protease and phosphatase inhibitors 1 ml buffer (biotool, Houston, TX, USA, 510019, B15001‐A, B15001‐B, B14001). After incubation on ice for 1 hr, the lysates were sonicated for 15 min. centrifuged at 12,000x *g* at 4°C, supernatant was collected. The supernatant was analysed for protein concentration using Bradford method 26. The absorbance recorded on a micro‐plate reader (Thermo Max micro‐plate reader; Molecular Devices, Sunnyvale, CA, USA). For Western blot analysis, 20 μg of protein extracted from NTM cells were mixed with Laemmli buffer (Bio‐Rad, Hercules, CA, USA) containing 0.1% β‐mercaptoethanol, 95°C for 5 min. and separated on a 10% SDS‐PAGE gel. Proteins were transferred to nitrocellulose membrane and probed with primary antibodies against β‐Catenin (Cells Signalling Technology, Danvers, MA, USA D10A8 XP ^R^ rabbit mAb, c8480s, 1:3000 dilution), pGSK‐3β (Cells Signalling Technology Ser9 5B3, D85E12, XP ^R^ rabbit mAb, 1:3000 dilution). β‐Actin was determined using anti‐β‐Actin (MP Biomedicals, Santa Ana, CA, USA, 691001, 1:15,000 dilution). Membranes were incubated for 1 hr in room temperature with secondary antibodies (Cells Signalling Technology anti‐rabbit IgG HRP‐linked antibody or antimouse Jackson Immuno Research). The immunocomplexes were detected with chemiluminescence reagent (Thermo Fisher, Pierce™ ECL Western Blotting Substrate) for 1 min., followed by exposure to Kodak X‐ray film (Rochester, NY, USA). Semiquantitative analysis was carried out using a computerized image analysis system, EZQuant Gel software (EZQuant Biology Solution, Tel‐Aviv, Israel). The results from replicate experiments converted to percentages of control by average.

### Gel Zymography

Matrix metalloproteinases (MMPs) are involved in NTM extracellular matrix metabolism and have been shown to increase aqueous out‐flow facility 16. Our purpose was to characterize effects of different NPCE‐derived EVs concentrations, on the activity of MMP2 and MMP9 in cultured human NTM cells. To do so, NTM cells were seeded at density of 2 × 10^6^ cells in 6 mm Petri dishes. After 48 hrs, cells were exposed to different NPCE EVs concentrations of (X1, X2 or X10) for 8 hrs. Next, the NTM cells were lysed with buffer containing 1 M HEPES (pH 7.4), 2 M NaCl, 100 mM EGTA, 100 mM EDTA, 10% glycerol, 10 mM MgCl_2_, 100% Triton x‐10 and protease and phosphatase inhibitors. After incubation on ice for 1 hr, the lysates were sonicated for 15 min. centrifuged at 12,000x *g* at 4°C, supernatant was collected. The supernatant was analysed for protein concentration using Bradford method. The absorbance recorded on a micro‐plate reader (Thermo Max micro‐plate reader; Molecular Devices). For Gel Zymography analysis, 20 μg of protein extracted from NTM cells were mixed with sample buffer consisting of 0.25 M Tris HCl pH 6.8, 40% glycerol, 8% SDS, 0.006% bromophenol blue, and were separated on a 10% SDS‐PAGE gel which was prepared with 1 mg/ml gelatin, at 150 V for 90 min. Each zymogram was washed in a renature buffer (2.5% Triton x‐100 solution) for 30 min. After removing renature buffer, developing buffer (50 mM Tris pH 7.5, 0.2 M NaCl, 5 mM CaCl_2_, 0.02% brij 35 at pH 7.6) was added, and after 30 min. on a 50 RPM on shaker, it was replaced with fresh one. The gel was incubated at 37°C for 8 hrs to examine optimal MMP's activity. After 8 hrs, developing buffer was replaced with 0.5% coomassie blue G‐250 in methanol/acetic acid/H_2_O (3:1:6). The stained gel was washed with DDW for 20 min. in room temperature and then incubated with fresh DDW overnight. Destaining buffer (45% ethanol, 5% glycerol, 50% H_2_O) was used and MMP's activity was analysed by Image J software.

### Statistics

Data are presented as mean ± standard deviation. Statistical evaluation of one‐way anova was performed with GraphPad Prism version 7.0 software (La Jolla, CA, USA). Differences between groups were tested using Tukey's test. The analysis of EVs labelling uptake by NTM cells along various concentrations was performed on the median values. All tests were considered significant at *P* < 0.05.

## Results

To achieve our aim to clarify physiological the ratio/proportion between NPCE‐derived EVs and their target cells, trabecular meshwork NTM, we used various quantifying techniques

### TRPS

**Figure 1:**
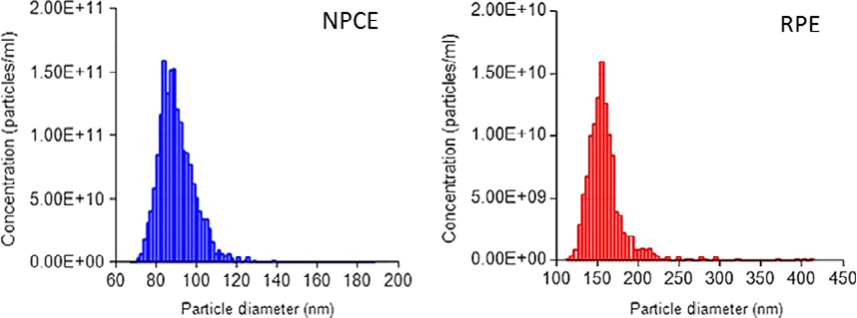
Size distribution of NPCE and RPE cell‐derived EVs as measured by Tunable Resistive Pulse Sensing (TPRS).

The size and concentration of EVs isolated from NPCE cell culture supernatant were measured using TRPS method (Fig. 1). EVs concentrations were within the range of 1 × 10^10^ to 1 x 10^12^ particles/ml, were size measured resulted in of 97.50 ± 10.21 and 128.33 ± 11.12 for NPCE‐ and RPE‐derived EVs, respectively. The size distribution for NPCE‐derived EVs was of 52–140 and 69–162 nm for RPE‐derived EVs.

### Image stream

A possible pathway of communication between NPCE cells and NTM cells in the drainage system of the eye is by uptake of EVs by the target cells 12. EVs secreted from NPCE cells are assumed to be released to the ocular anterior and posterior chambers, through the aqueous humour, and finally enter into NTM cells 13.

**Figure 2:**
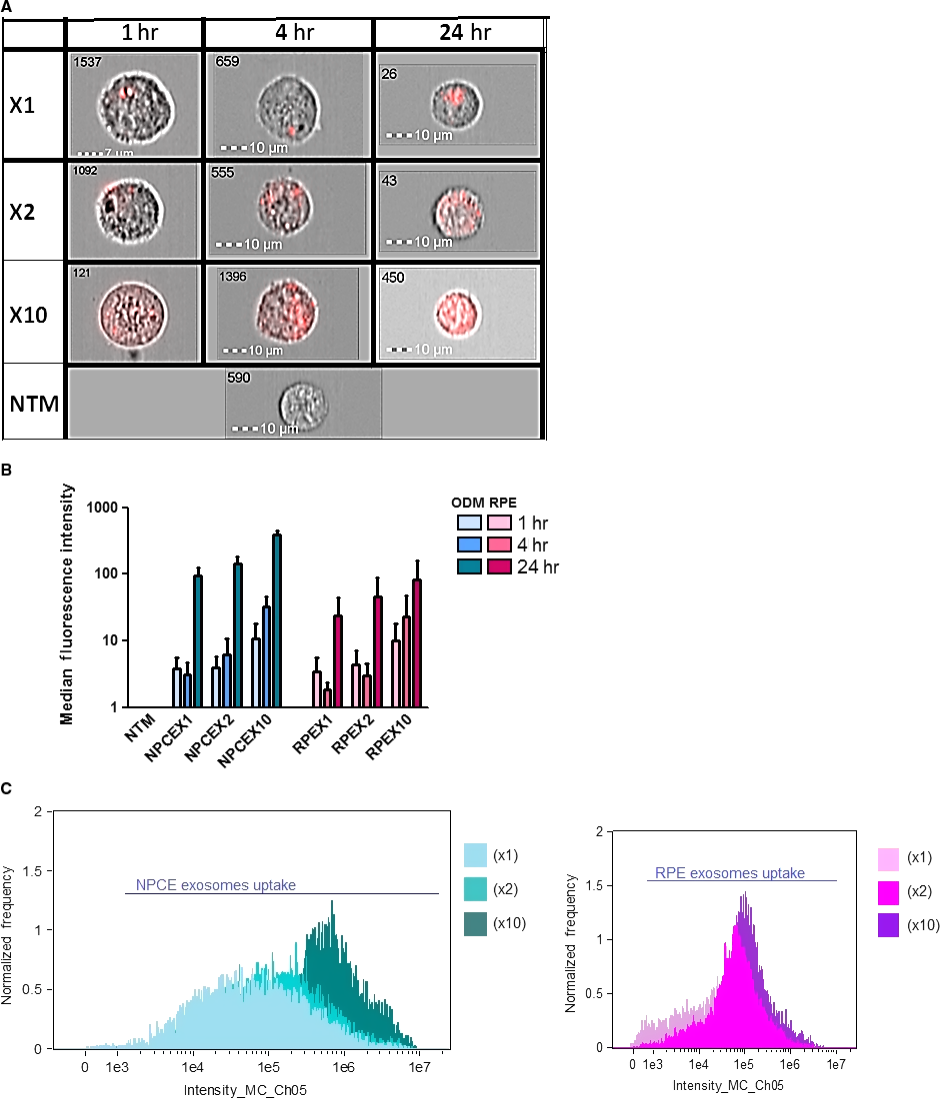
NPCE‐derived EVs in several concentrations (X1, X2 or X10) were stained with DiD for three different incubation times, and a dose–response uptake by NTM cells was found. The left corner numbers are the event number out of 10,000 events (**A**). NPCE‐ and RPE‐derived EVs uptake after 1, 4 or 24 hrs of incubation with NTM cells were analysed by Image Stream. A significant (*P* < 0.05) increase in NPCE EVs uptake was found as a function of on EVs concentrations (**B**). NTM cell fluorescence due to DiD‐labelled NPCE‐ or RPE‐derived EVs were monitored (**C**). Data represent means ± S.D from three independent experiments performed in triplicates.

To examine the effect of various EVs concentrations on the signalling transference between the NPCE and the NTM cells, DiD‐stained EVs accumulation in a NTM cell culture was analysed at 1, 4 and 24 hrs 27. The results (Fig. 2A) show that EVs (red) were found inside the NTM cells in all three time‐points. The ImageStream measurements indicated a correlation between the level of target cells EVs accumulation as a function of concentrations (Fig. 2B). Mean fluorescence intensity measurements indicate that there is a specificity of the cellular uptake of NPCE‐derived EV by NTM cells as compared to RPE‐derived EV (Fig. 2C).

The viability of treated NTM cells with various EVs concentrations as compared to untreated NTM cells was examined using 7AAD fluorescence dye. The overall viability was not affected by EVs incubation for 24 hrs and may not represent an adequate biological significance. 99.9% of the NTM cells were negative for 7AAD were NPCE‐derived EVs‐treated NTM cells were negative, 99.96% for X1, X2 and X10, for 7AAD fluorescence, respectively. RPE‐derived EVs‐treated NTM cells were negative for 7AAD; 99.95%, 99.97% and 99.96% for X1, X2 and X10 respectively. Data are representative of two independent experiments, and the number of captured events was 10,000 NTM cells of each sample.

### RT‐PCR

**Figure 3:**
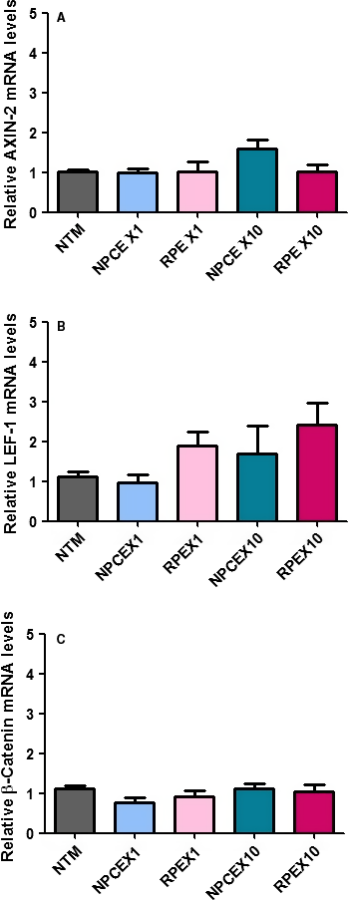
Real‐time qRT‐PCR was performed to examine the mRNA expression levels of canonical WNT/β‐ catenin signalling components, including the Wnt transducers Axin2 (**A**), LEF1 (**B**) and β‐ catenin (**C**). Data were normalized to the average mRNA level of GADPH. One‐way anova analyses were used to study the effect of different exosome concentrations on Wnt genes expression. Data represent means ± S.D from three independent experiments performed in triplicates.

A potential pathway involved in the effects of NPCE‐derived EVs on NTM cells is the canonical Wnt‐Akt‐TGFβ2 signalling which leads to β‐catenin activation 28. Thus, we investigated whether the exposure of NTM cells to different NPCE‐derived EVs concentrations affects the endogenous expression of Wnt‐regulated genes. We examined the expression of: β‐catenin, AXIN2 and LEF1 genes for possible up‐regulation at various EVs concentrations, by tracking their specific mRNA levels. Real‐Time PCR analysis revealed that treatment of NTM cells with high concentration (X10) of NPCE‐derived EV for 2 hrs showed a trend of increase in AXIN2 and LEF1 expression *versus* untreated control or *versus* low EV concentrations (X1). RPE‐derived EVs exhibited an increase LEF1 mRNA expression when low (X1) and high (X10) concentrations were used (Fig. 3).

### Western blot

**Figure 4:**
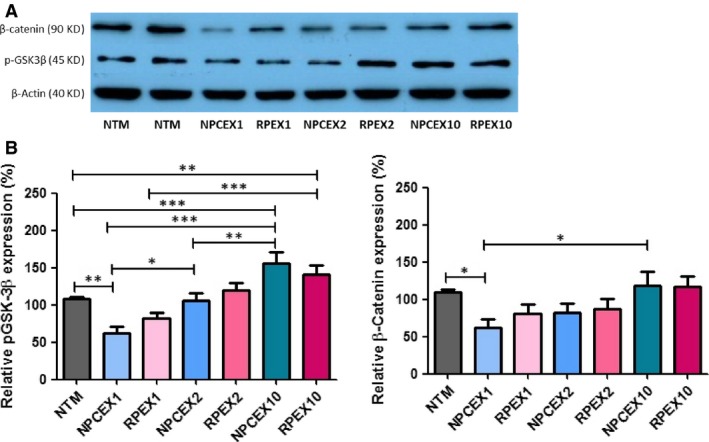
β‐Catenin and pGSK‐3β proteins levels normalized to β‐Actin (40 KD) after 8 hrs of incubation in NTM cells following exposure to different concentrations of ODM and RPE EVs. Total protein was extracted from NTM cells and analysed by Western blot. (**A**) Representative Western blot film. (**B**) A significant increase in β‐Catenin (90 KD) expression in NTM cells following (X10) treatment of NPCE EVs. A significant increase in pGSK‐3β (45 KD) expression in NTM cells following (X10) and (X2) treatments of NPCE EVs. The bar graph represents the means ± S.D from three independent experiments performed in triplicates. One‐way ANOVA was used to determine statistical difference, as indicated by asterisks (*P < 0.05, **P < 0.01, ***P < 0.001).

To evaluate the effect of EVs dose–response in NPCE‐NTM communication, confluent NTM cells were exposed for 8 hrs to various concentrations of NPCE‐derived EVs. RPE‐derived EVs were used as control (Fig. 4). We focused on NTM expression of GSK3‐β and β‐catenin, previously reported to be affected by NPCE‐derived EVs treatment 12. Exposure to low EVs concentration was associated with decreased expression of β‐Catenin (*P* < 0.05) and pGSK‐3β (*P* < 0.01), as compared to untreated NTM cells used as control. Our results demonstrate that in high EVs concentration (×10), the decreased expression of β‐Catenin (*P* < 0.05) and pGSK‐3β (*P* < 0.001) found for low concentrations was even diminished as compared to untreated control.

### Gel Zymography

**Figure 5:**
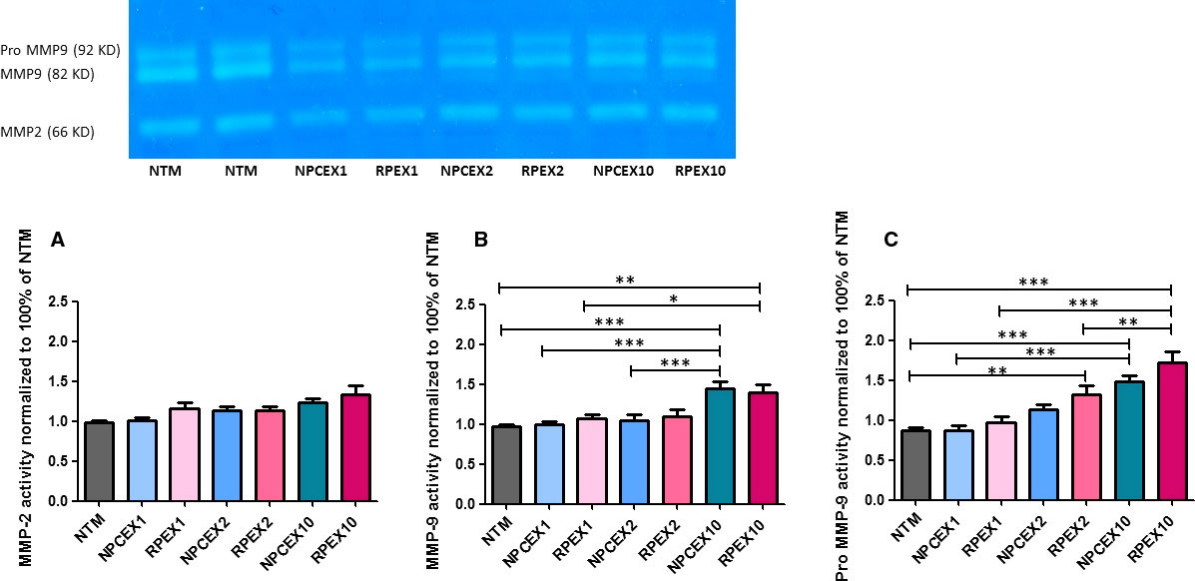
(**A**) MMP2 (**B**) MMP9 (**C**) Pro‐MMP9 quantitative analysis of NTM cells in coculture with NPCE EV or RPE EV for 8 hrs. Representative MMP's activity relative to NTM cells (untreated). Data represent means ± S.D from three independent experiments performed in triplicates. One‐way ANOVA was used to determine statistical difference, as indicated by asterisks (*P < 0.05, **P < 0.01, ***P < 0.001).

To investigate the effect of various NPCE‐derived EV concentrations on MMP's activity in NTM cells, gelatin zymography was performed. Activity of Pro‐MMP9, MMP9 and MMP2 was detected as bands on zymograms (Fig. 5). MMPs activity of cocultured TM cells with various concentrations of NPCE EV was compared to untreated control NTM cells, or to coculture with various concentrations of RPE EV as control for the specificity of the interaction. All experimental groups showed the presence of pro‐MMP9, MMP9 and MMP2. Pro‐MMP2 activity was not detected in any of the treatments. Three independent experiments yielded similar results showing that pro‐MMP9 and MMP9 activity was significantly enhanced in NTM cells treated with high EV concentrations of (×10) as compared to low EV concentrations of either NPCE‐ or RPE‐derived EVs and to untreated control. MMP2 activity showed the same trend of increase, but did not reach significance (Fig. 5).

## Discussion

Over the last years, EVs have received considerable attention suggesting their role as signalling mediators. EVs cargo includes the following: signal proteins, lipids, DNA and mirRNA that are in part characteristic to the paternal cells they shed from. Consequently, EVs are capable of delivering complex information to target cells.The present study explored NPCE‐derived EVs dose–response effects on NTM cells. We were able to show that exposure to different NPCE EVs concentrations result in a different response of NTM canonical Wnt signalling. As customary in the literature, our *in vitro* experiments were conducted by incubating a determined amount of EVs with the target cells. What should be this “determined amount of EVs”? Sverdlov in his paper questioned the physiologically relevance of EVs used in *in vitro* experiments and expressed concern about using dozens to hundreds micrograms of EVs 29. EVs dose–response literature is limited so far. A bimodal dose‐dependent paracrine effect of tumour EVs on endothelium morphological changes *in vitro* was reported using a range of 0–10 μg/ml. The authors have suggested that the differential response to tumour EVs is attributable to different signalling at different EVs dose 29.

In our study, expression changes in two key canonical Wnt signalling proteins were detected as follows: pGSK3β and β‐catenin. The phosphorylation of GSK3β allows the release of dephosphorylated β‐catenin from its inactive complex, to translocate into the cell nucleus were it can induce translation of mRNA involved in cell adhesion such as; LEF1, Axin2 and β‐catenin. We showed that when a relatively low number of NPCE‐derived EVs were incubated with NTM cells, a significant decrease in the expression of pGSK3 and β‐catenin was found. In our study, 6.6 × 10^9^ EVs (as counted by TPRS method) were incubated with 2.0 × 10^6^ NTM cells. This amount of EVs (X1) contained 2 μg EVs proteins, a relatively low EVs:Cell ratio. Incubating the NTM cells with higher EVs amount (X2) = 13.6 × 10^9^ or (X10) = 6.8 × 10^10^ for the same 8 hrs incubation time resulted in the abolishment of the down‐regulation expression induced on NTM pGSK3β and β‐catenin. Bimodal dose dependence is a known effect in pharmacology and was reported for oestrogen, cocaine, alcohol and other drugs, and also for cytokines: IL‐1, IL‐4, growth factors and second messengers. Bimodal effects by EVs make sense considering the diversity of possible signalling molecules and signalling pathways that have been assigned to them 25. Under our EVs, target cells communication experiments, a significant EVs dose dependent increase in the proteins expression was detected. The present study found that low EVs dose decrease protein expression *versus* control while higher EVs dose increase protein expression resemble those of Hood *et al*.

Comparing our experiments to other published EVs research that used similar protocols, places the various EVs doses used in the present study in the lower range. Our initial experiments using the X1 EVs dose were performed along the lower EVs protein content used in Hood *et al*. uptake studies 17. The experiments with higher EVs amount (X10) containing 20 μg proteins is similar to the EVs protein content reported by Tian *et al*. 30. However, other researchers have used X10 and even higher EVs concentrations 31, 32.

The mechanism of uptake of NPCE‐derived EV by NTM cells is yet unknown, and is presently assumed to be related to partially specific diffusion or uptake 12, as opposed to specific EVs recognition by a membrane transporter 33. The effect of NPCE‐derived EVs on NTM cells in our study was specific to the expression of Wnt signalling pathway components. Other biological effects including related gene expression and downstream MMPs activity were found to exhibit a general dose–response effect. A trend in the gene expression changes detected as early as 2 hrs of incubation was reported to be significant at low EVs amount (X1) following 4 hrs of EVs incubation with the NTM cells 12, earlier to the pGSK3β and β‐catenin changes at 8 hrs of EVs incubation. We hypothesize that a direct effect like a ligand binding to the Wnt receptor on NTM cells might result in the gene expression changes while the protein expression changes at 8 hrs of EVs incubation can reflect endocytosis processes. Data obtained by ImageStream analysis supported the entry of the EVs as fast as 8 hrs of incubation. In our previous report, 4 hrs of NPCE incubation at X1 resulted in a beginning of EVs accumulation within the NTM target cell by 4 hrs, reaching a maximum at 12 hrs 12.

However, limitations of our study should be acknowledged. The EVs dosing concentrations may not be sufficient for analysing a full dose–response. The use of cell lines for EVs source and targets need validations assays in primary cells model.

In conclusion, our data support the concept that EVs biological effects are concentration‐dependent at their target site. Specifically in the present study, we described a general dose–response at the gene and MMPs activity and a different response regarding key canonical Wnt proteins expression depending on EVs doses.

## Conflict of interest

The authors have declared that no competing interests exist.
